# Ethane-1,2-diaminium 4,4′-sulfonyl­dibenzoate

**DOI:** 10.1107/S1600536811041274

**Published:** 2011-10-12

**Authors:** Graham Smith, Urs D. Wermuth

**Affiliations:** a Faculty of Science and Technology, Queensland University of Technology, GPO Box 2434, Brisbane, Queensland 4001, Australia

## Abstract

In the title salt, C_2_H_10_N_2_^2+^·C_14_H_8_O_6_S^2−^, both the ethyl­ene­diaminium cations and the 4,4′-sulfonyl­dibenzoate dianions have crystallographic twofold rotational symmetry. They are inter­linked by aminium N—H⋯O_carboxyl­ate_ hydrogen-bonding associations, giving sheets parallel to (101) and are further linked along [010], forming a three-dimensional structure.

## Related literature

For the structure of 4,4′-sulfonyl­dibenzoic acid, see: Lian *et al.* (2007 ▶). For the structures of some metal complexes of the acid, see: Bannerjee *et al.* (2009 ▶); Jiao (2010 ▶); Pan *et al.* (2007 ▶); Wu *et al.* (2007 ▶); Zhuang & Jin (2007 ▶).
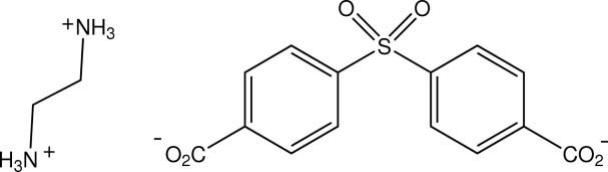


## Experimental

### 

#### Crystal data

C_2_H_10_N_2_^2+^·C_14_H_8_O_6_S^2−^
*M* *_r_* = 366.39
Monoclinic, 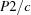

*a* = 15.2860 (8) Å
*b* = 4.8436 (2) Å
*c* = 11.9803 (6) Å
β = 111.812 (6)°
*V* = 823.51 (8) Å^3^
*Z* = 2
Mo *K*α radiation
μ = 0.23 mm^−1^
*T* = 200 K
0.35 × 0.25 × 0.08 mm

#### Data collection

Oxford Diffraction Gemini-S CCD detector diffractometer
Absorption correction: multi-scan (*CrysAlis PRO*; Oxford Diffraction, 2010 ▶) *T*_min_ = 0.98, *T*_max_ = 0.99
5062 measured reflections
1607 independent reflections
1290 reflections with *I* > 2σ(*I*)
*R*_int_ = 0.024

#### Refinement

*R*[*F* ^2^ > 2σ(*F* ^2^)] = 0.034
*wR*(*F* ^2^) = 0.098
*S* = 1.05
1607 reflections
114 parameters
H-atom parameters constrained
Δρ_max_ = 0.35 e Å^−3^
Δρ_min_ = −0.25 e Å^−3^

### 

Data collection: *CrysAlis PRO* (Oxford Diffraction, 2010 ▶); cell refinement: *CrysAlis PRO*; data reduction: *CrysAlis PRO*; program(s) used to solve structure: *SIR92* (Altomare *et al.*, 1994 ▶); program(s) used to refine structure: *SHELXL97* (Sheldrick, 2008 ▶) within *WinGX* (Farrugia, 1999 ▶); molecular graphics: *PLATON* (Spek, 2009 ▶); software used to prepare material for publication: *PLATON*.

## Supplementary Material

Crystal structure: contains datablock(s) global, I. DOI: 10.1107/S1600536811041274/lh5347sup1.cif

Structure factors: contains datablock(s) I. DOI: 10.1107/S1600536811041274/lh5347Isup2.hkl

Supplementary material file. DOI: 10.1107/S1600536811041274/lh5347Isup3.cml

Additional supplementary materials:  crystallographic information; 3D view; checkCIF report

## Figures and Tables

**Table 1:** Hydrogen-bond geometry (Å, °)

*D*—H⋯*A*	*D*—H	H⋯*A*	*D*⋯*A*	*D*—H⋯*A*
N1*A*—H11*A*⋯O42^i^	0.89	1.87	2.760 (2)	174
N1*A*—H12*A*⋯O41^ii^	0.89	1.88	2.740 (2)	163
N1*A*—H13*A*⋯O42	0.89	1.93	2.798 (2)	164

Symmetry codes: (i) 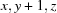; (ii) 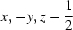.

**Fig. 1.**
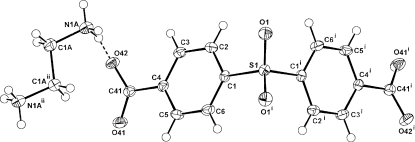
Molecular conformation and atom-numbering scheme for the title compound, with the inter-species hydrogen bond shown as a dashed line and with non-H atoms shown as 50% probability displacement ellipsoids. Both the dication and the dianion have twofold rotational symmetry [symmetry codes (i) -x + 2, y, -z + 3/2; (ii) -x + 1, y, -z + 1/2].

**Fig. 2.**
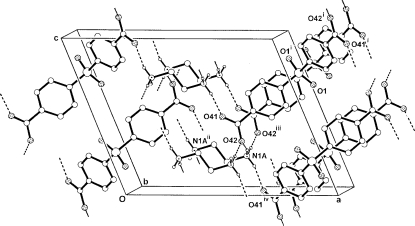
A perspective view of the three-dimensional structure looking along the b axial direction, showing hydrogen-bonding associations as dashed lines. Carbon-bound H atoms are omitted. For symmetry codes, see Table 1 and Fig. 1.

## supplementary crystallographic information

### Comment

The structure of the diprotic acid 4,4'-sulfonyldibenzoic acid (SDBA) is known (Lian *et al.*, 2007) and although some metal complexes with SDBA alone have been reported, *e.g.* with Li (Bannerjee *et al.*, 2009), Zn (Pan *et al.*, 2007; Zhuang & Jin, 2007) and Cd (Jiao, 2010), most structures have been with mixed ligands including this acid (Wu *et al.*, 2007). No structures of compounds of SDBA with Lewis bases are known. Our 1:1 stoichiometric reaction of this acid with ethylenediamine gave the the title compound C_2_H_10_N_2_^2+^ C_14_H_8_O_6_S^2-^, and the structure is reported here. In this structure (Fig. 1), both the ethylenediaminium cations and the 4,4'-sulfonyldibenzoate dianions have crystallographic twofold rotational symmetry. In contrast, the two substituted ring systems of the parent molecule are mirror related (Lian *et al.*, 2007). With the present salt, the central C1—S1—C1^i^ bond angle is 104.90 (8)Å [for symmetry code (i), see Fig. 1], while the carboxyl group (defined by atoms C4–C41–O41–O42) lies slightly out of the plane of the benzene ring [dihedral angle 19.31 (9)°]. The ethylenediamine cation is essentially planar [torsion angle N1A—C1A—C1A^ii^—N1A^ii^, 171.97 (14)°]. For symmetry code (ii), see also Fig. 1.

Intermolecular cation aminium *N*—*H*···O_carboxyl_ hydrogen bonds (Table 1) interlink the SDBA dianions into sheets lying in the (101) planes, as well as down the *b* axis, forming a three-dimensional structure (Fig. 2). The sulfonyl O atoms are involved in inter-species C—H···O associations [C2—H2···O1^v^, 3.200 (2) Å: symmetry code (v) -*x* + 2, -*y* + 1, -*z* + 1]. as well as in S—O···*Cg* interactions [minimum S1—O1···*Cg*(ring C1^viii^–C6^viii^) = 3.5409 (13) Å; S—O···*Cg* angle, 90.61 (5)°: symmetry code (viii) -*x* + 2, *y* + 1, -*z* + 3/2].

### Experimental

The title compound was synthesized by heating together for 10 min under reflux, 1 mmol quantities of 4,4'-sulfonyldibenzoic acid and ethylenediamine in 50 ml of 50% ethanol–water. After evaporation of the solvent the non-crystalline product was recrystallized from a 50% methanol–isopropyl alcohol solution giving thin colourless crystal plates from which a specimen was cleaved for the X-ray analysis..

### Refinement

The aminium H atoms were located by difference Fourier methods and their positional and isotropic displacement parameters were initially refined but finally were allowed to ride on the N atom with *U*_iso_(H) = 1.2*U*_eq_(N). Other H atoms were included at calculated positions with C—H (aromatic) = 0.93 Å or C—H (methyl) = 0.97 Å] and also treated as riding, with *U*_iso_(H) = 1.2*U*_eq_C(aromatic) or 1.5*U*_eq_C(methylene).

### Crystal data

**Table:**

C_2_H_10_N_2_^2+^·C_14_H_8_O_6_S^2^^−^	*F*(000) = 384
*M**_r_* = 366.39	*D*_x_ = 1.478 Mg m^−^^3^
Monoclinic, *P*2/*c*	Mo *K*α radiation, λ = 0.71073 Å
Hall symbol: -P 2yc	Cell parameters from 2936 reflections
*a* = 15.2860 (8) Å	θ = 3.5–28.6°
*b* = 4.8436 (2) Å	µ = 0.23 mm^−^^1^
*c* = 11.9803 (6) Å	*T* = 200 K
β = 111.812 (6)°	Plate, colourless
*V* = 823.51 (8) Å^3^	0.35 × 0.25 × 0.08 mm
*Z* = 2	

### Data collection

**Table:**

Oxford Diffraction Gemini-S CCD detector diffractometer	1607 independent reflections
Radiation source: Enhance (Mo) X-ray source	1290 reflections with *I* > 2σ(*I*)
graphite	*R*_int_ = 0.024
Detector resolution: 16.077 pixels mm^-1^	θ_max_ = 26.0°, θ_min_ = 3.4°
ω scans	*h* = −18→18
Absorption correction: multi-scan (*CrysAlis PRO*; Oxford Diffraction, 2010)	*k* = −5→5
*T*_min_ = 0.98, *T*_max_ = 0.99	*l* = −14→14
5062 measured reflections	

### Refinement

**Table:**

Refinement on *F*^2^	Primary atom site location: structure-invariant direct methods
Least-squares matrix: full	Secondary atom site location: difference Fourier map
*R*[*F*^2^ > 2σ(*F*^2^)] = 0.034	Hydrogen site location: inferred from neighbouring sites
*wR*(*F*^2^) = 0.098	H-atom parameters constrained
*S* = 1.05	*w* = 1/[σ^2^(*F*_o_^2^) + (0.0627*P*)^2^] where *P* = (*F*_o_^2^ + 2*F*_c_^2^)/3
1607 reflections	(Δ/σ)_max_ < 0.001
114 parameters	Δρ_max_ = 0.35 e Å^−^^3^
0 restraints	Δρ_min_ = −0.25 e Å^−^^3^

### Special details

**Table:**

Geometry. Bond distances, angles *etc*. have been calculated using the rounded fractional coordinates. All su's are estimated from the variances of the (full) variance-covariance matrix. The cell e.s.d.'s are taken into account in the estimation of distances, angles and torsion angles
Refinement. Refinement of *F*^2^ against ALL reflections. The weighted *R*-factor *wR* and goodness of fit *S* are based on *F*^2^, conventional *R*-factors *R* are based on *F*, with *F* set to zero for negative *F*^2^. The threshold expression of *F*^2^ > σ(*F*^2^) is used only for calculating *R*-factors(gt) *etc*. and is not relevant to the choice of reflections for refinement. *R*-factors based on *F*^2^ are statistically about twice as large as those based on *F*, and *R*- factors based on ALL data will be even larger.

### Fractional atomic coordinates and isotropic or equivalent isotropic displacement parameters (Å^2^)

**Table:**

	*x*	*y*	*z*	*U*_iso_*/*U*_eq_	
S1	1.00000	0.61975 (12)	0.75000	0.0180 (2)	
O1	1.03086 (9)	0.7681 (3)	0.66702 (11)	0.0249 (4)	
O41	0.63470 (9)	−0.2635 (3)	0.51471 (11)	0.0301 (4)	
O42	0.66855 (9)	−0.1734 (3)	0.35316 (10)	0.0243 (4)	
C1	0.90679 (11)	0.3967 (3)	0.66620 (15)	0.0175 (5)	
C2	0.90534 (12)	0.2888 (4)	0.55790 (16)	0.0225 (5)	
C3	0.83192 (12)	0.1147 (4)	0.49238 (15)	0.0221 (5)	
C4	0.76174 (11)	0.0437 (4)	0.53517 (14)	0.0176 (5)	
C5	0.76491 (13)	0.1538 (4)	0.64426 (16)	0.0216 (5)	
C6	0.83708 (12)	0.3320 (4)	0.71004 (16)	0.0221 (5)	
C41	0.68270 (12)	−0.1474 (3)	0.46402 (15)	0.0192 (5)	
N1A	0.61734 (10)	0.3312 (3)	0.23264 (13)	0.0242 (5)	
C1A	0.51341 (13)	0.3110 (4)	0.19485 (16)	0.0273 (6)	
H2	0.95280	0.33250	0.52980	0.0270*	
H3	0.82960	0.04480	0.41900	0.0260*	
H5	0.71830	0.10750	0.67330	0.0260*	
H6	0.83850	0.40660	0.78220	0.0260*	
H11A	0.63740	0.48570	0.27460	0.0290*	
H12A	0.63270	0.33380	0.16790	0.0290*	
H13A	0.64420	0.18630	0.27810	0.0290*	
H14A	0.48380	0.46620	0.14350	0.0330*	
H15A	0.49130	0.14250	0.14940	0.0330*	

### Atomic displacement parameters (Å^2^)

**Table:**

	*U*^11^	*U*^22^	*U*^33^	*U*^12^	*U*^13^	*U*^23^
S1	0.0190 (3)	0.0165 (3)	0.0174 (3)	0.0000	0.0055 (2)	0.0000
O1	0.0270 (7)	0.0217 (7)	0.0255 (7)	−0.0024 (5)	0.0091 (6)	0.0053 (6)
O41	0.0338 (8)	0.0381 (8)	0.0204 (7)	−0.0146 (6)	0.0123 (6)	−0.0022 (6)
O42	0.0310 (7)	0.0265 (7)	0.0153 (6)	−0.0061 (5)	0.0086 (5)	−0.0033 (5)
C1	0.0170 (8)	0.0169 (9)	0.0167 (8)	0.0018 (6)	0.0041 (7)	0.0021 (7)
C2	0.0214 (9)	0.0292 (10)	0.0195 (9)	−0.0025 (7)	0.0107 (7)	−0.0014 (8)
C3	0.0257 (9)	0.0265 (10)	0.0158 (9)	−0.0013 (8)	0.0098 (7)	−0.0031 (7)
C4	0.0177 (8)	0.0187 (9)	0.0148 (8)	0.0021 (7)	0.0041 (7)	0.0028 (7)
C5	0.0237 (9)	0.0244 (10)	0.0214 (9)	−0.0038 (7)	0.0140 (8)	−0.0008 (7)
C6	0.0268 (9)	0.0244 (10)	0.0169 (9)	−0.0014 (8)	0.0103 (8)	−0.0037 (7)
C41	0.0215 (9)	0.0185 (9)	0.0179 (9)	0.0027 (7)	0.0077 (7)	0.0027 (7)
N1A	0.0292 (9)	0.0254 (8)	0.0189 (8)	−0.0004 (7)	0.0099 (7)	−0.0003 (7)
C1A	0.0245 (10)	0.0361 (11)	0.0209 (10)	0.0020 (8)	0.0080 (8)	−0.0024 (8)

### Geometric parameters (Å, °)

**Table:**

S1—O1	1.4407 (14)	C2—C3	1.390 (3)
S1—C1	1.7726 (17)	C3—C4	1.393 (3)
S1—O1^i^	1.4407 (14)	C4—C41	1.509 (2)
S1—C1^i^	1.7726 (17)	C4—C5	1.396 (2)
O41—C41	1.247 (2)	C5—C6	1.392 (3)
O42—C41	1.270 (2)	C2—H2	0.9300
N1A—C1A	1.485 (3)	C3—H3	0.9300
N1A—H13A	0.8900	C5—H5	0.9300
N1A—H11A	0.8900	C6—H6	0.9300
N1A—H12A	0.8900	C1A—C1A^ii^	1.522 (3)
C1—C2	1.391 (2)	C1A—H14A	0.9700
C1—C6	1.388 (3)	C1A—H15A	0.9700
			
O1—S1—C1	108.27 (8)	C4—C5—C6	120.77 (18)
O1—S1—O1^i^	120.17 (9)	C1—C6—C5	118.97 (17)
O1—S1—C1^i^	107.12 (8)	O42—C41—C4	116.17 (16)
O1^i^—S1—C1	107.12 (8)	O41—C41—O42	124.21 (16)
C1—S1—C1^i^	104.90 (8)	O41—C41—C4	119.61 (15)
O1^i^—S1—C1^i^	108.27 (8)	C3—C2—H2	121.00
C1A—N1A—H11A	109.00	C1—C2—H2	120.00
C1A—N1A—H12A	110.00	C2—C3—H3	120.00
C1A—N1A—H13A	109.00	C4—C3—H3	120.00
H11A—N1A—H12A	109.00	C4—C5—H5	120.00
H11A—N1A—H13A	109.00	C6—C5—H5	120.00
H12A—N1A—H13A	109.00	C5—C6—H6	120.00
C2—C1—C6	121.32 (16)	C1—C6—H6	121.00
S1—C1—C2	119.21 (14)	N1A—C1A—C1A^ii^	109.76 (15)
S1—C1—C6	119.47 (13)	N1A—C1A—H14A	110.00
C1—C2—C3	118.96 (17)	N1A—C1A—H15A	110.00
C2—C3—C4	120.83 (16)	H14A—C1A—H15A	108.00
C3—C4—C41	120.53 (15)	C1A^ii^—C1A—H14A	110.00
C3—C4—C5	119.13 (17)	C1A^ii^—C1A—H15A	110.00
C5—C4—C41	120.35 (16)		
			
O1—S1—C1—C2	30.61 (16)	C2—C3—C4—C5	1.2 (3)
O1—S1—C1—C6	−149.72 (14)	C2—C3—C4—C41	−179.47 (17)
O1^i^—S1—C1—C2	161.56 (14)	C3—C4—C5—C6	−0.1 (3)
O1^i^—S1—C1—C6	−18.77 (16)	C41—C4—C5—C6	−179.47 (17)
C1^i^—S1—C1—C2	−83.51 (15)	C3—C4—C41—O41	162.10 (17)
C1^i^—S1—C1—C6	96.15 (15)	C3—C4—C41—O42	−19.4 (2)
S1—C1—C2—C3	−179.80 (14)	C5—C4—C41—O41	−18.5 (3)
C6—C1—C2—C3	0.5 (3)	C5—C4—C41—O42	159.93 (17)
S1—C1—C6—C5	−179.17 (14)	C4—C5—C6—C1	−0.7 (3)
C2—C1—C6—C5	0.5 (3)	N1A—C1A—C1A^ii^—N1A^ii^	171.97 (14)
C1—C2—C3—C4	−1.4 (3)		

Symmetry codes: (i) −*x*+2, *y*, −*z*+3/2; (ii) −*x*+1, *y*, −*z*+1/2.

### Hydrogen-bond geometry (Å, °)

**Table:**

*D*—H···*A*	*D*—H	H···*A*	*D*···*A*	*D*—H···*A*
N1A—H11A···O42^iii^	0.89	1.87	2.760 (2)	174
N1A—H12A···O41^iv^	0.89	1.88	2.740 (2)	163
N1A—H13A···O42	0.89	1.93	2.798 (2)	164
C2—H2···O1^v^	0.93	2.51	3.200 (2)	131
C5—H5···O42^vi^	0.93	2.56	3.344 (2)	143
C6—H6···O1^i^	0.93	2.55	2.915 (2)	104
C1A—H14A···O41^vii^	0.97	2.46	3.384 (2)	160

Symmetry codes: (iii) *x*, *y*+1, *z*; (iv) *x*, −*y*, *z*−1/2; (v) −*x*+2, −*y*+1, −*z*+1; (vi) *x*, −*y*, *z*+1/2; (i) −*x*+2, *y*, −*z*+3/2; (vii) −*x*+1, *y*+1, −*z*+1/2.
